# Scientific Notes

**Published:** 1883-02

**Authors:** 


### SCIENTIFIC NOTES.

In making steel, it is important to get rid of the phosphorus. Krupp & Bell first melt the iron with 15 to 20 per cent of oxide of iron in a rotating oven, thus eliminating three-quarters of the phosphorus.

Dr. Duryee's theory—which his experiments seem to prove—is that in his revolving furnace, with his blow-pipe flame, fed with chloride of sodium, the phosphorus is eliminated in the shape of a chloride, when the heat is over 3,000 degrees F.

As the metal melts, carbon, sulphur, and other hurtful matters pass out, leaving a refined iron. At this stage, any desired grade of steel or malleable iron can be made, by the addition of manganese or pieces of iron. He has lately made important experiments in the puddling and conversion of iron. In the reduction of sulphuret ores, not a trace of sulphur was found after running through his furnace. We believe that he has solved the problem of the treatment of rebellious ores, whether gold, silver, or copper. When it is considered that three-quarters of all the precious metals are locked up in sulphur, and that no ordinary heat will release them, and, further, that as large a proportion of iron is contaminated with phosphorus and other substances fatal to the manufacture of steel, we can appreciate what we believe he has accomplished for some of the great industries of the country, by the invention and practical application of his ingenious blow-pipe furnace with its irresistible heat.

